# Influence of low‐dose aspirin, resistance exercise, and sex on human skeletal muscle PGE_2_/COX pathway activity

**DOI:** 10.14814/phy2.14790

**Published:** 2021-03-04

**Authors:** Masatoshi Naruse, William A. Fountain, Alex Claiborne, Toby L. Chambers, Andrew M. Jones, Andrew M. Stroh, Cristhian F. Montenegro, Colleen E. Lynch, Kiril Minchev, Scott Trappe, Todd A. Trappe

**Affiliations:** ^1^ Human Performance Laboratory Ball State University Muncie IN USA

**Keywords:** cyclooxygenase, low‐dose aspirin, prostaglandin E_2_, resistance exercise, skeletal muscle

## Abstract

Prostaglandin (PG) E_2_ has been linked to increased inflammation and attenuated resistance exercise adaptations in skeletal muscle. Nonaspirin cyclooxygenase (COX) inhibitors have been shown to reduce these effects. This study examined the effect of low‐dose aspirin on skeletal muscle COX production of PGE_2_ at rest and following resistance exercise. Skeletal muscle (vastus lateralis) biopsies were taken from six individuals (4 M/2 W) before and 3.5 hr after a single bout of resistance exercise for *ex vivo* PGE_2_ production under control and low (10 μM)‐ or standard (100 μM)‐dose aspirin conditions. Sex‐specific effects of aspirin were also examined by combining the current findings with our previous similar *ex vivo* skeletal muscle investigations (n = 20, 10 M/10 W). Low‐dose aspirin inhibited skeletal muscle PGE_2_ production (*p* < 0.05). This inhibition was similar to standard‐dose aspirin (*p* > 0.05) and was not influenced by resistance exercise (*p* > 0.05) (overall effect: −18 ± 5%). Men and women had similar uninhibited skeletal muscle PGE_2_ production at rest (men: 1.97 ± 0.33, women: 1.96 ± 0.29 pg/mg wet weight/min; *p* > 0.05). However, skeletal muscle of men was 60% more sensitive to aspirin inhibition than women (*p* < 0.05). In summary, the current findings 1) confirm low‐dose aspirin inhibits the PGE_2_/COX pathway in human skeletal muscle, 2) show that resistance exercise does not alter aspirin inhibitory efficacy, and 3) suggest the skeletal muscle of men and women could respond differently to long‐term consumption of low‐dose aspirin, one of the most common chronically consumed drugs in the world.

## Graphical abstract

Low‐dose aspirin can significantly reduce PGE_2_/COX pathway activity in human skeletal muscle, and this inhibitory effect of aspirin is not altered by resistance exercise. The skeletal muscle of men is significantly more sensitive than women to aspirin inhibition of this inflammation‐regulating pathway. These findings have implications for the skeletal muscle health of sedentary and resistance exercise training men and women that regularly consume low‐dose aspirin.

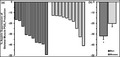

## 1 INTRODUCTION

Aspirin is a potent cyclooxygenase (COX) inhibitor that is commonly consumed for its anti‐inflammatory and cardioprotective effects (O'Brien et al., 2019; Stuntz & Bernstein, 2017; Zhou et al., 2014). Recent investigations have shown that aspirin inhibits COX production of the inflammatory regulator prostaglandin E_2_ (PGE_2_) in resting human skeletal muscle under *in vivo* and *ex vivo* conditions (Fountain et al., 2020; Ratchford et al., 2017). Considering that aspirin is consumed chronically at low doses by an estimated 65 million adults in the United States, it is noteworthy that low‐dose aspirin significantly reduces skeletal muscle PGE_2_ production (Fountain et al., 2020).

Regular consumption of nonaspirin COX inhibitors positively impacts skeletal muscle mass in sedentary individuals, as well as in individuals undergoing resistance exercise training (Beyer et al., 2011; Landi et al., 2013; Rieu et al., 2009; Trappe et al., 2011, 2016). Numerous cellular responses regulate the skeletal muscle adaptations to resistance exercise (Adams & Bamman, 2012; Egan & Zierath, 2012), and PGs produced through skeletal muscle COX have been shown to be involved in this regulation (Trappe & Liu, 2013). In particular, PGE_2_, the most abundant PG produced in skeletal muscle, regulates skeletal muscle protein metabolism and inflammation through its autocrine and paracrine influence on myocellular and molecular processes, ultimately impacting skeletal muscle mass and function (Ho et al., 2017; Korotkova & Lundberg, 2014; Liu et al., 2016; Schaap et al., 2009; Standley et al., 2013; Trappe & Liu, 2013; Trappe et al., 2013). In addition, resistance exercise results in unique alterations in the intracellular environment (Adams & Bamman, 2012; Egan & Zierath, 2012; Powers et al., 2016), which may influence COX enzyme function or the efficacy of drugs that inhibit COX (Feldman et al., 2000; Liu et al., 2016; Ratchford et al., 2017; Simmons et al., 2004; Smith & Malkowski, 2019; Smith et al., 2011; Trappe & Liu, 2013). Yet, the potential influence of aspirin on skeletal muscle PGE_2_/COX pathway activity after resistance exercise is unknown.

There are established sex‐based differences in the pharmacokinetics of aspirin (Ho et al., 1985; Kelton et al., 1981; Menguy et al., 1972; Miaskiewicz et al., 1982; Miners et al., 1986). Specifically, absorption and clearance appear to be influenced, but these studies do not provide skeletal muscle‐specific information. As there are few investigations into the effects of aspirin on skeletal muscle (Fountain et al., 2020; Ratchford et al., 2017), any potential sex‐specific effects are unknown. Lack of sex‐specific information is not uncommon in drug development, and there are likely more differences in drug responsiveness between men and women than has been previously appreciated (Zucker & Prendergast, 2020).

The purpose of the current investigation was to determine whether we could replicate the findings of low‐dose aspirin inhibition of the PGE_2_/COX pathway in human skeletal muscle (Fountain et al., 2020) and to determine whether the inhibitory efficacy was influenced by resistance exercise. *Ex vivo* skeletal muscle incubation was utilized to directly examine drug–tissue interactions and to eliminate confounding issues associated with systemic drug absorption, clearance, and tissue delivery (Roden & George, 2002; Rowland et al., 2011). We hypothesized that low‐dose aspirin concentrations would suppress skeletal muscle PGE_2_ production and that low‐dose aspirin efficacy would be reduced following resistance exercise. An additional exploratory objective was to examine potential sex‐specific effects of aspirin on skeletal muscle by combining the data from the current and previous (Fountain et al., 2020; Ratchford et al., 2017) investigations, which used the same methodologies to examine the PGE_2_/COX pathway activity in skeletal muscle.

## 2 METHODS

### 2.1 Subjects

All subjects were physically active (i.e., regular aerobic and/or resistance exercise 3–5 days/week), nonsmokers, and apparently healthy. None of the subjects chronically consumed prescription or nonprescription analgesic or anti‐inflammatory drugs. All study procedures, risks, and benefits were explained to the subjects before giving written consent to participate. This study was approved by the Institutional Review Board of the Ball State University.

Subjects underwent a dual‐energy X‐ray absorptiometry (DXA) scan (Lunar iDXA; GE Healthcare, Madison WI) for body composition assessment. Subjects also performed a continuous cycle ergometer (Lode Excalibur Sport; Lode BV, Groningen, Netherlands) test with 12‐lead ECG (ST80i; Philips Medical Systems, Andover, MA) to volitional exhaustion for the determination of maximal oxygen consumption (VO_2_max). Oxygen uptake was determined every 30 seconds through an automated open‐circuit indirect calorimetry system incorporating electronic O_2_ and CO_2_ analyzers (S‐3A/I and CD‐3A; AEI Technologies, Pittsburgh, PA). The gas analyzers were calibrated with gases of known concentration. Subjects completed a 2‐minute warm‐up (men: 100 W, women: 50 W) followed by a ramped increase in the workload (men: 25 W/min, women: 20 W/min) until the subjects reached volitional fatigue. Successful testing criteria included a plateau in the volume of oxygen consumed (VO_2_), a respiratory exchange ratio of ≥1.10, and a rating of perceived exertion ≥19. Subject characteristics of the individuals (4 M/2 W) included in this investigation are presented in Table 1. Subjects also completed resistance exercise familiarization prior to an acute resistance exercise trial with muscle biopsies (Fountain et al., 2020; Sanford et al., 2020). Specific pretrial controls were in place for COX inhibitor consumption, diet, physical activity, and menstrual cycle timing for the women. Details regarding the resistance exercise familiarization and acute resistance exercise trial, including the pretrial controls, are presented below.

**TABLE 1:** Subject characteristics

Variable	
n	6 (4 M, 2 W)
Age, y	25 ± 1
Height, cm	176 ± 4
Weight, kg	81.5 ± 3.4
BMI, kg/m^2^	26 ± 1
Body fat, %	29 ± 4
VO_2_max, L/min	3.45 ± 0.29
VO_2_max, ml/kg/min	42.8 ± 3.1
Leg press 1RM, kg	149 ± 24
Knee extension 1RM, kg	117 ± 13

Data are mean ± SE.

### 2.2 Resistance exercise familiarization

Subjects underwent three familiarization sessions, each separated by at least 48 hours. The purpose of the familiarization sessions was to determine the 10 repetition maximum (RM) for each of the exercises to be used during the acute resistance exercise trial: chest press, overhead press, seated row, triceps extension, biceps curl, leg press, leg curl, and knee extension. Each session started with a 5‐minute warm‐up on a cycle ergometer (828E; Monark Exercise AB, Vansbro, Sweden). The first familiarization session established the individual settings and proper form for each exercise on the cable motion strength equipment (Life Fitness, Rosemont, IL) at a light intensity. The second and third familiarization sessions consisted of the subjects performing one or two sets, respectively, to volitional fatigue on each exercise. The resistances used during the third familiarization session were determined based on the performance of the previous familiarization sessions, so subjects would reach exhaustion at repetition 10 of each set. Strength (1RM) of the subjects was also determined for the quadriceps‐focused exercises (leg press and knee extension) during the first and second familiarizations (Table 1).

### 2.3 Acute resistance exercise trial

**FIGURE 1:**
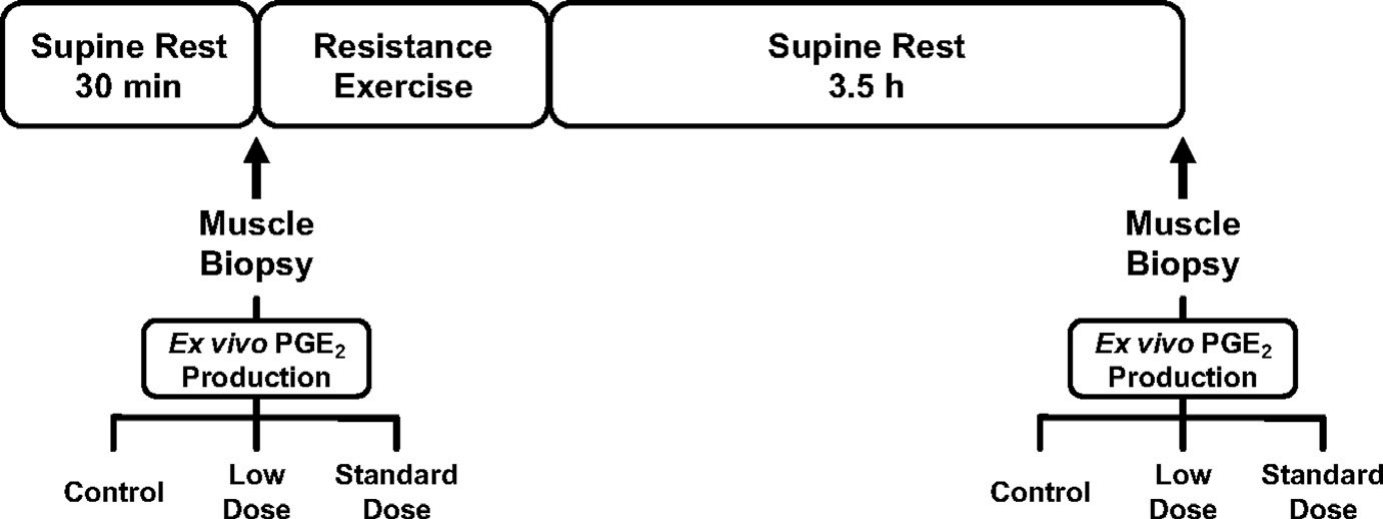
Protocol schematic for the acute resistance exercise trial with vastus lateralis muscle biopsies taken before and after exercise for *ex vivo* incubation measurements of skeletal muscle PGE_2_ production. For each muscle biopsy, three separate muscle strips were immediately placed in Krebs–Henseleit buffer (control) supplemented with one of two different aspirin concentrations that reflected blood levels following a low (10 µM; typical oral dose: 75–325 mg) or standard (100 µM; typical oral dose: 975–1000 mg) dose. Further details are provided in the Methods section

Acute resistance exercise trials were completed at least 3 days after the last familiarization session in the morning after an overnight fast. Each trial consisted of a supine rest period of at least 30 minutes prior to a baseline muscle biopsy, a resistance exercise bout followed by a 3.5‐hour supine rest period, and a postexercise muscle biopsy (Figure 1). The postexercise timepoint was chosen because of the increased metabolic and molecular activity related to exercise adaptation (Sanford et al., 2020) and the increased COX activity and PG production (Carroll et al., 2013; Trappe & Liu, 2013; Vella et al., 2019). In addition, this timepoint coincided with our previous study on aspirin and aerobic exercise (Fountain et al., 2020).

#### 2.3.1 Pretrial controls

Prior to the trial, subjects were instructed to maintain normal dietary habits while refraining from 1) COX inhibitor (e.g., aspirin, acetaminophen, ibuprofen) consumption for 7 days, 2) alcohol consumption and exercise training for 48 hours, and 3) caffeine consumption for 24 hours. The evening before the trial, subjects were instructed to consume their evening meal no later than 7 pm and a liquid nutritional supplement (Ensure Plus; Abbott Laboratories, Columbus, OH; 8 oz, 350 kcal, 57% carbohydrate, 15% protein, and 28% fat) 10 hr prior to the scheduled initial muscle biopsy the following morning. This supplement allowed for the final nutrient intake and fast duration to be standardized across subjects. Only water was allowed after consumption of the standardized nutrition until the completion of the trial the next day. All women completed the acute resistance exercise trial between days 3 and 7 of their menstrual cycle.

#### 2.3.2 Resistance exercise bout

Subjects completed a 5‐minute warm‐up on a cycle ergometer, followed by 3 sets of the familiarized resistance exercises to volitional fatigue (~10 repetitions) with 90‐second rest between each set. The leg press, leg curl, and knee extension were completed as the final three exercises, so the quadriceps (vastus lateralis)‐focused exercise was completed last prior to the controlled rest period and postexercise muscle biopsy.

#### 2.3.3 Muscle biopsy

Subjects underwent a skeletal muscle biopsy of the vastus lateralis following local anesthetic (lidocaine HCl 1%) with a 6‐mm Bergström needle (Bergström, 1962) with suction before (resting) and 3.5 hr after exercise (Figure 1). One biopsy was performed on each leg. Following each muscle biopsy, excess blood, visible fat, and connective tissue were removed, and the muscle was divided and processed for the *ex vivo* incubation (Figure 1).

### 2.4 Ex Vivo Skeletal Muscle Incubation and PGE_2_ Analysis

These procedures have been performed in previous studies from our laboratory (Fountain et al., 2020; Ratchford et al., 2017). Following the biopsy, each of the three muscle strips (18.0 ± 0.9 mg) was immediately placed into separate incubation vials containing 2 ml of pregassed (95% O_2_ / 5% CO_2_) Krebs–Henseleit buffer (KHB) (118.5 mM NaCl, 1.2 mM MgSO_4_, 4.7 mM KCl, 1.2 mM KH_2_PO_4_, 25 mM NaHCO_3_, 2.5 mM CaCl_2_, pH 7.4) supplemented with 5 mM glucose. One vial contained only this gassed KHB solution (control), while the two additional vials also contained either 10 µM (low dose) or 100 µM (standard dose) aspirin (Sigma A2093) (Figure 1). An aspirin stock solution (1 mM) was made fresh immediately prior to each trial using reagent grade water (James, 1958), before adding it to the KHB. All vials were kept at room temperature for 10 minutes prior to incubation at 37°C with constant agitation under an atmosphere of 95% O_2_ / 5% CO_2_ for 20 minutes (total preincubation period of 30 minutes). Each sample was then transferred to new vials containing 2 ml of fresh pregassed KHB, KHB+10 µM aspirin, or KHB+100 µM aspirin. Additionally, each of these new vials contained 5 µM arachidonic acid (BML‐FA003‐0100; Enzo Life Sciences, Farmingdale, NY). These vials were incubated for an additional 30 minutes at 37°C with constant agitation under an atmosphere of 95% O_2_ / 5% CO_2_. At the end of the 30‐minute incubation period, the muscle samples were frozen and stored in liquid nitrogen and the incubation media samples were stored at −80°C until analysis.

The arachidonic acid concentration was chosen to replicate our previous *ex vivo* investigations of human vastus lateralis muscle at rest and following aerobic exercise in men and women (Fountain et al., 2020; Ratchford et al., 2017). This concentration was also chosen because it provides a linear PG production rate for ≥60 min (Fagan & Goldberg, 1986); it does not saturate the COX enzyme in human skeletal muscle (Ratchford et al., 2017); it coincides with reported *K*_m_ values in isolated human COX enzymes (Smith et al., 2011; Tsai & Kulmacz, 2010); it stimulates PGE_2_ production and protein turnover in incubated animal muscle, and these responses can be blunted by aspirin (Rodemann & Goldberg, 1982); and it stimulates PGE_2_ production in isolated human skeletal muscle by a magnitude that is observed *in vivo* in response to exercise (Boushel et al., 2002; Ratchford et al., 2017; Trappe et al., 2001).

All incubation media samples used for determination of PGE_2_ production in the presence of 5 µM arachidonic acid, with and without aspirin, were removed from −80°C and thawed at room temperature. Samples were analyzed for PGE_2_ in triplicate (K051‐H5; Arbor Assays, Ann Arbor, MI), and sample concentrations were determined using a 4PLC curve based on PGE_2_ standards diluted in KHB. Each incubated skeletal muscle strip was removed from liquid nitrogen and weighed at ~‐24°C (Cahn C‐35; Orion Research, Beverly, MA). This weight was used in the calculation of PGE_2_ production over the 30‐minute incubation period.

### 2.5 Sex‐specific comparisons

Data from the current investigation and two previous investigations that focused on aspirin and skeletal muscle (Fountain et al., 2020; Ratchford et al., 2017) were combined to allow for a larger sample size comparison of men and women. These three investigations used similar subject populations, pretrial controls (COX inhibitor consumption, diet, physical activity, and the timing of the menstrual cycle for the women), muscle biopsy sampling and processing, *ex vivo* incubation procedures, and PGE_2_ assay procedures. As a result, 10 men (26 ± 1 y, 183 ± 3 cm, 86.1 ± 2.5 kg) and 10 women (24 ± 0.5 y, 166 ± 2 cm, 68.1 ± 3.0 kg) that were responsive to aspirin were compared for aspirin inhibition of skeletal muscle PGE_2_ production.

### 2.6 Statistics

Pre‐ and postexercise skeletal muscle PGE_2_ production responses for the three aspirin conditions (control, low dose, and standard dose) were compared with a two‐way (exercise and aspirin) analysis of variance (ANOVA) with repeated measures. Control and aspirin responses, independent of dose and exercise, were compared with a paired t test. Skeletal muscle PGE_2_ production and aspirin responses between men and women, independent of dose and exercise, were compared with an unpaired t test. Significance was accepted at *p* < 0.05. Values are presented as mean ± SE.

## 3 RESULTS

Subjects averaged 10 repetitions per set for the leg press and 10 repetitions per set for the knee extension, for a total of 60 repetitions focused on the quadriceps (vastus lateralis). Average load during the three sets of leg press (104 ± 17 kg) and knee extension (76 ± 8 kg) was 70% and 65% of the 1RM for these two exercises, respectively.

**FIGURE 2:**
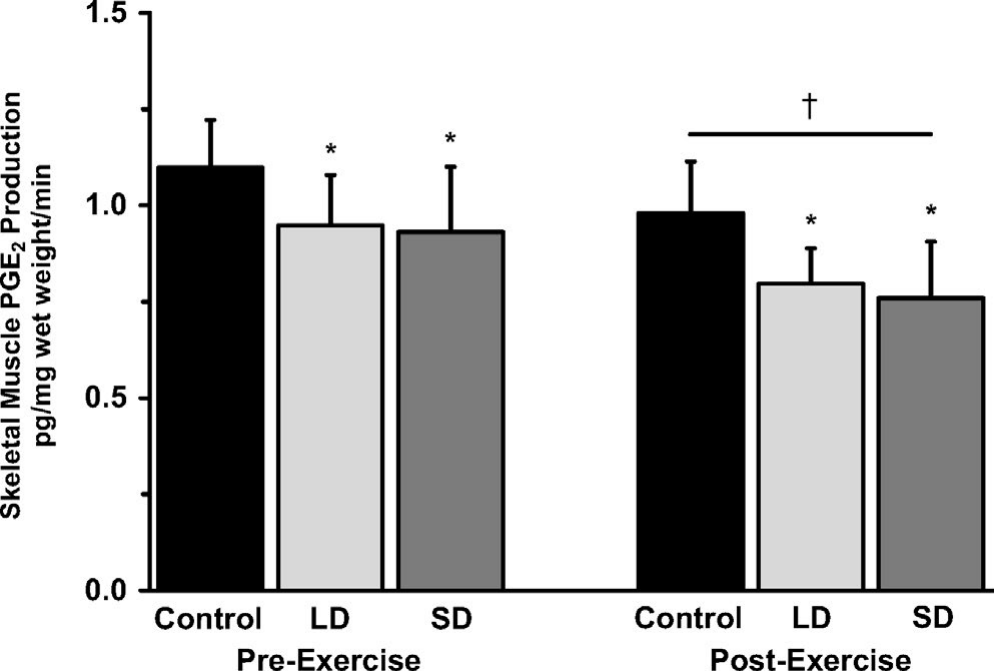
The influence of aspirin dose and resistance exercise on *ex vivo* skeletal muscle PGE_2_ production. Data are from the 6 subjects in the current study. *p < 0.05 for aspirin (LD: low dose, SD: standard dose) vs. control, independent of dose and exercise. †*p* < 0.05 for resistance exercise vs. pre‐exercise, independent of aspirin condition

Weights of the incubated vastus lateralis muscle strips were similar across the control (19.3 ± 1.1 mg), low‐dose aspirin (18.2 ± 1.5 mg), and standard‐dose aspirin (16.6 ± 1.9 mg) conditions. Skeletal muscle PGE_2_ production in the presence of 5 µM arachidonic acid and the various aspirin conditions from muscle biopsies taken before and after exercise are presented in Figure 2. Low‐ and standard‐dose aspirin similarly (*p* > 0.05) reduced muscle PGE_2_ production (−18 ± 5%; *p* < 0.05), independent of resistance exercise. Resistance exercise decreased PGE_2_ production 3.5 hr postexercise (−15 ± 5%; *p* < 0.05), independent of aspirin condition. Individual responsiveness to low‐ and standard‐dose aspirin suppression of PGE_2_ ranged from −10 to −47%, with one subject who did not respond to aspirin across the two doses (−2%).

**FIGURE 3:**
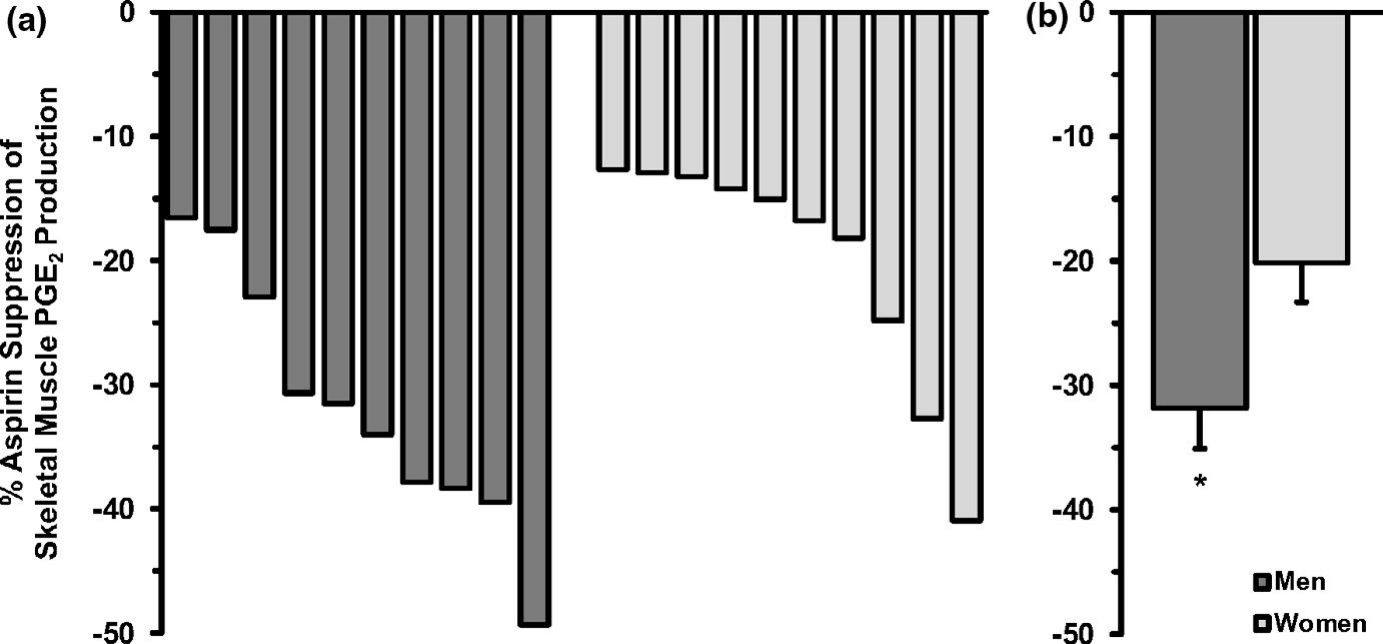
Sex‐specific aspirin suppression of *ex vivo* skeletal muscle PGE_2_ production. Data are a compilation of men (n = 10) and women (n = 10) that responded to aspirin and were studied using similar methodologies (current study, Fountain et al., 2020; Ratchford et al., 2017). Further details are provided in the *Sex*‐*Specific Comparisons* section of the Methods. (a): Individual responses of the men and women, from lowest to highest suppression. Study designation (current study^a^, Fountain et al., 2020 ^b^, Ratchford et al., 2017 ^c^): For the men, left to right: a, c, a, b, b, c, c, b, a, c; For the women, left to right: a, c, c, a, c, b, b, b, b, c. (b): Average suppression of the men and women. *p < 0.05 vs. women

For the sex‐specific comparisons, skeletal muscle PGE_2_ production under resting control conditions (without aspirin) was similar (*p* > 0.05) between men (1.97 ± 0.33 pg/mg wet weight/min) and women (1.96 ± 0.29 pg/mg wet weight/min). However, aspirin reduced muscle PGE_2_ production by 60% more in men compared with women (*p* < 0.05; Figure 3). Individual responsiveness to aspirin suppression in the men and women is also presented in Figure 3.

## 4 DISCUSSION

The current investigation focused on the PGE_2_/COX pathway because of its well‐known influence on myocellular regulation and adaptations, as well as the known impact of nonaspirin COX‐inhibiting drugs (Ho et al., 2017; Korotkova & Lundberg, 2014; Schaap et al., 2009; Standley et al., 2013; Trappe & Liu, 2013; Trappe et al., 2016). The focus was also on aspirin because it is one of the most commonly consumed drugs in the world. The current results confirm that low‐dose aspirin can significantly inhibit COX in skeletal muscle and reduce the inflammatory and skeletal muscle health regulator PGE_2_ (Fountain et al., 2020). The relative efficacy of low‐dose aspirin on skeletal muscle PGE_2_ production has been surprising. Interestingly, similar effectiveness of low‐dose aspirin compared with doses up to eight times higher has been shown in nonskeletal muscle tissue (Sample et al., 2002). The current results also extend our previous findings on aerobic exercise (Fountain et al., 2020) to include resistance exercise and thus encompass the two most common forms of exercise recommended to the general population for skeletal muscle and overall health (Piercy et al., 2018; Sanford et al., 2020). Finally, as data start to build on aspirin and skeletal muscle, it appears that aspirin has a sex‐specific effect on the PGE_2_/COX pathway at the skeletal muscle tissue level.

The lack of resistance exercise reducing aspirin efficacy was contrary to our hypothesis and suggests aspirin acetylation of COX in human skeletal muscle is independent of typical exercise‐induced changes in the myocellular environment (Adams & Bamman, 2012; Egan & Zierath, 2012; Powers et al., 2016; Simmons et al., 2004; Smith & Malkowski, 2019; Smith et al., 2011). This is similar to what was observed at the same timepoint following a standard bout of aerobic exercise (Fountain et al., 2020), which has different changes in the myocellular environment that lead to different phenotypic adaptations (Adams & Bamman, 2012; Egan & Zierath, 2012; Powers et al., 2016). Translation of these acute exercise response findings into studies of individuals regularly performing resistance or aerobic exercise is needed.

Interestingly, the resistance exercise bout decreased arachidonic acid‐stimulated skeletal muscle PGE_2_ production and this was unexpected. Skeletal muscle biopsy measurements of intramuscular PGE_2_ levels generally show an increase in the first 24 hr following resistance exercise (Trappe et al., 2001, 2002; Vella et al., 2019). However, measurements of skeletal muscle interstitial PGE_2_ levels via microdialysis do not confirm the biopsy obtained increases (Mikkelsen et al., 2008; Paulsen et al., 2010). The discrepancies across studies could be related to the sampling technique and/or the wide variety of resistance exercise approaches used in these studies. In addition, the COX produced precursor to all prostaglandins, PGH_2_, is elevated in skeletal muscle biopsy tissue four hr after resistance exercise (Carroll et al., 2013). Biopsy and microdialysate levels of other PGs (F_2α_) in skeletal muscle have also been shown to be increased in the first 24 hr following resistance exercise (Trappe et al., 2001, 2006; Vella et al., 2019). Thus, PG production through the COX enzyme and downstream synthases is generally increased in skeletal muscle for up to a day following a single session of resistance exercise. Yet, it is not clear why the addition of the 5 µM arachidonic acid to the *ex vivo* incubation did not stimulate skeletal muscle PGE_2_ production 3.5 hr following the resistance exercise bout. We can speculate that synthesis of the terminal PGs (E_2_, F_2α_, D_2_, I_2_) may be regulated independently by the downstream synthases that generate and interconvert the specific PGs from PGH_2_ (Liu et al., 2016; Smith et al., 2011; Trappe & Liu, 2013). Some of these specific enzymes have been studied and shown to be variably influenced by acute and chronic resistance exercise (Lavin et al., 2020a; Trappe et al., 2013). We also cannot rule out the regulation of *in vivo* arachidonic acid flux and other intracellular factors (Adams & Bamman, 2012; Egan & Zierath, 2012; Irvine, 1982; Powers et al., 2016; Smith & Malkowski, 2019; Smith et al., 2011) that may influence the PGE_2_/COX pathway after resistance exercise.

The current and previous (Fountain et al., 2020; Ratchford et al., 2017) studies collectively provide unique insight into sex‐specific differences in the aspirin inhibitory influence on the PGE_2_/COX pathway in skeletal muscle. While the skeletal muscle tissue of men was more sensitive than women to aspirin inhibition of skeletal muscle PGE_2_ production (Figure 3), reasons for this tissue‐specific sex difference are not readily apparent. The main reported sex‐specific differences related to aspirin metabolism center on pharmacokinetics and tissue delivery (Ho et al., 1985; Kelton et al., 1981; Menguy et al., 1972; Miaskiewicz et al., 1982; Miners et al., 1986). These reported differences would have been eliminated with the *ex vivo* approach used for these studies. Unfortunately, other COX inhibitor studies do not provide sex‐specific skeletal muscle information or insight into the potential differences between men and women. Although speculative, inherent sex‐specific abundance differences in the PGE_2_/COX pathway substrate or enzymes (i.e., COX, cPGES, mPGES‐1, or mPGES‐2; (Liu et al., 2016)) could have played a role. Future studies are clearly warranted to better understand this apparent skeletal muscle‐specific effect that could have implications for millions of men and women that consume low‐dose aspirin chronically.

Several other results from the current investigation align with previous findings (Fountain et al., 2020; Ratchford et al., 2017). The amount of aspirin inhibition using the same *ex vivo* incubation model has been similar across all three studies, and the individual variation to aspirin inhibition has also been consistent. Given the tissue‐specific nature of the *ex vivo* measurement, this might be explained by the individual profile of skeletal muscle PGE_2_/COX pathway components (Liu et al., 2016; Trappe & Liu, 2013), which is known to be influenced by skeletal muscle fiber type (Liu et al., 2016; Trappe et al., 2016). The consistent proportion of skeletal muscle aspirin‐resistant individuals is in general agreement with the clinically observed aspirin resistance related to coagulation (Campbell et al., 2005; Hovens et al., 2007). However, the underlying basis for aspirin resistance in skeletal muscle is unknown and needs further investigation.

### 4.1 Limitations and future directions

We only focused on the PGE_2_ branch of the COX pathway because of the strong evidence of the importance of this PG from resting, acute and chronic exercise, and mechanistic molecular studies (Ho et al., 2017; Karamouzis, Karamouzis, et al., 2001; Karamouzis, Langberg, et al., 2001; Korotkova & Lundberg, 2014; Lavin et al., 2020a, 2020b; Liu et al., 2016; Rodemann & Goldberg, 1982; Standley et al., 2013; Trappe et al., 2001, 2011, 2013, 2016; Trappe & Liu, 2013; Vella et al., 2019). Measurements of other PGs from different branches of the COX pathway could be examined in future studies to provide supportive information (Smith et al., 2011; Trappe & Liu, 2013). Interrogation of transcriptional and translational regulatory mechanisms related to the effect of aspirin on skeletal muscle could also provide additional interesting scientific insight. The current incubation duration was not designed to specifically address these types of questions (Fiebich et al., 2001; Standley et al., 2013; Wang et al., 2010). Other timepoints following exercise would also add to our understanding, given the window of PGE_2_ production in skeletal muscle appears to be at least the first 24 hr after resistance exercise. While we are confident in the sex‐specific findings from across the three studies given the homogeneity of the methodologies and research team, larger scale studies should focus on discovering the underlying differences between men and women. The related investigations and the clear effect of aspirin on the PGE_2_/COX pathway in skeletal muscle suggest a wide variety of investigations should be undertaken to improve our understanding in this area.

## 5 CONCLUSIONS

This study furthers our understanding of the influence of aspirin in skeletal muscle inflammatory regulation through the PGE_2_/COX pathway. The current results confirm low‐dose aspirin could impact inflammatory‐related skeletal muscle health in sedentary individuals (Fountain et al., 2020) and extend these findings to resistance exercising individuals. It is also apparent that the effects of aspirin on skeletal muscle PGE_2_/COX pathway inhibition are sex‐specific, based on tissue‐specific differences between men and women. Further research is needed to identify the impacts of different types of exercise training on the PGE_2_/COX pathway in skeletal muscle of men and women and the potential interactions with chronic use of low‐dose aspirin on skeletal muscle health.

## CONFLICT OF INTEREST

No conflicts of interest, financial or otherwise, are declared by the authors.

## AUTHOR CONTRIBUTIONS

MN, WAF, AC, AMJ, ST, and TAT conceived and designed the research. MN, WAF, TLC, KM, and TAT analyzed the data. MN, WAF, AC, AMJ, and TAT interpreted the results of experiments. MN and TAT prepared the figures and drafted the manuscript. MN, WAF, AC, TLC, AMJ, AMS, CFM, CEL, KM, ST, and TAT performed the experiments, edited and revised the manuscript, and approved the final version of the manuscript.
